# Massive Mandibular Fibrous Dysplasia Mimicking Calcifying Epithelial Odontogenic Tumor Radiologically: A Case Report

**DOI:** 10.7759/cureus.113051

**Published:** 2026-07-20

**Authors:** Poosapati D Devasilpa Raju, Sudam Sadangi, Snehasis Pradhan, Priyadarshini Dehuri, Amit Samal, Sunil Agrawala

**Affiliations:** 1 Surgical Oncology, Institute of Medical Sciences and Sum Hospital, Bhubaneswar, IND; 2 Pathology, Institute of Medical Sciences and Sum Hospital, Bhubaneswar, IND

**Keywords:** differential diagnosis, fibro-osseous lesion, fibrous dysplasia, mandibular tumor, mandibulectomy, pindborg tumor

## Abstract

Fibrous dysplasia is a benign fibro-osseous lesion that may occasionally mimic odontogenic tumors radiologically. We report a rare case of a massive mandibular fibro-osseous lesion in a 58-year-old female presenting with progressively increasing lower facial swelling, trismus, and neck pain for four years. Contrast-enhanced computed tomography revealed a large (13 × 11 × 15 cm) expansile lytic lesion with dense calcified nodules, cortical thinning, and cortical breaches, radiologically suggestive of calcifying epithelial odontogenic tumor (Pindborg tumor). Initial biopsy demonstrated fibrocollagenous tissue with dead bone; however, due to the aggressive radiological appearance and extensive mandibular involvement, provisional diagnosis favored calcifying epithelial odontogenic tumor. The patient underwent subtotal mandibulectomy with reconstruction plate fixation, local flap reconstruction, and elective tracheostomy. Final histopathological examination revealed a fibro-osseous lesion with irregular immature bony trabeculae within fibrous stroma, consistent with fibrous dysplasia. Postoperatively, the patient was managed with regular oral cleaning, tracheostomy care, and speech and swallow therapy. This case highlights the diagnostic challenges associated with giant mandibular fibro-osseous lesions and emphasizes the importance of definitive histopathological evaluation.

## Introduction

Calcifying epithelial odontogenic tumor (CEOT), also known as Pindborg tumor, is a rare benign odontogenic neoplasm accounting for less than 1% of all odontogenic tumors [1]. It was first described by Pindborg in 1955 and commonly presents as a painless, slow-growing swelling involving the posterior mandible [2]. Radiographically, CEOT characteristically appears as a mixed radiolucent-radiopaque lesion with varying degrees of calcification [3]. Fibrous dysplasia, on the other hand, is a benign fibro-osseous lesion characterized by replacement of normal bone by immature woven bone within fibrous stroma. Although fibrous dysplasia typically demonstrates a ground-glass appearance radiologically, giant lesions with extensive calcification may occasionally mimic odontogenic tumors, leading to diagnostic difficulty. Herein, we present a rare case of massive mandibular fibrous dysplasia radiologically mimicking CEOT and discuss the diagnostic challenges, surgical management, and histopathological correlation.

## Case presentation

A 58-year-old female presented with a four-year history of progressively increasing lower facial swelling associated with trismus and progressive neck pain for three months. There was no history of dysphagia, foreign body sensation, thyroid disorder, or other major comorbidities. Her body mass index was 13.8 kg/m², and her body weight was 32 kg, likely secondary to chronic difficulty with oral intake. Clinical examination revealed a massive hard, non-ulcerated swelling involving the mandible with significant facial deformity (Figure 1). No cervical lymphadenopathy or cranial nerve deficit was identified. 

**Figure 1:**
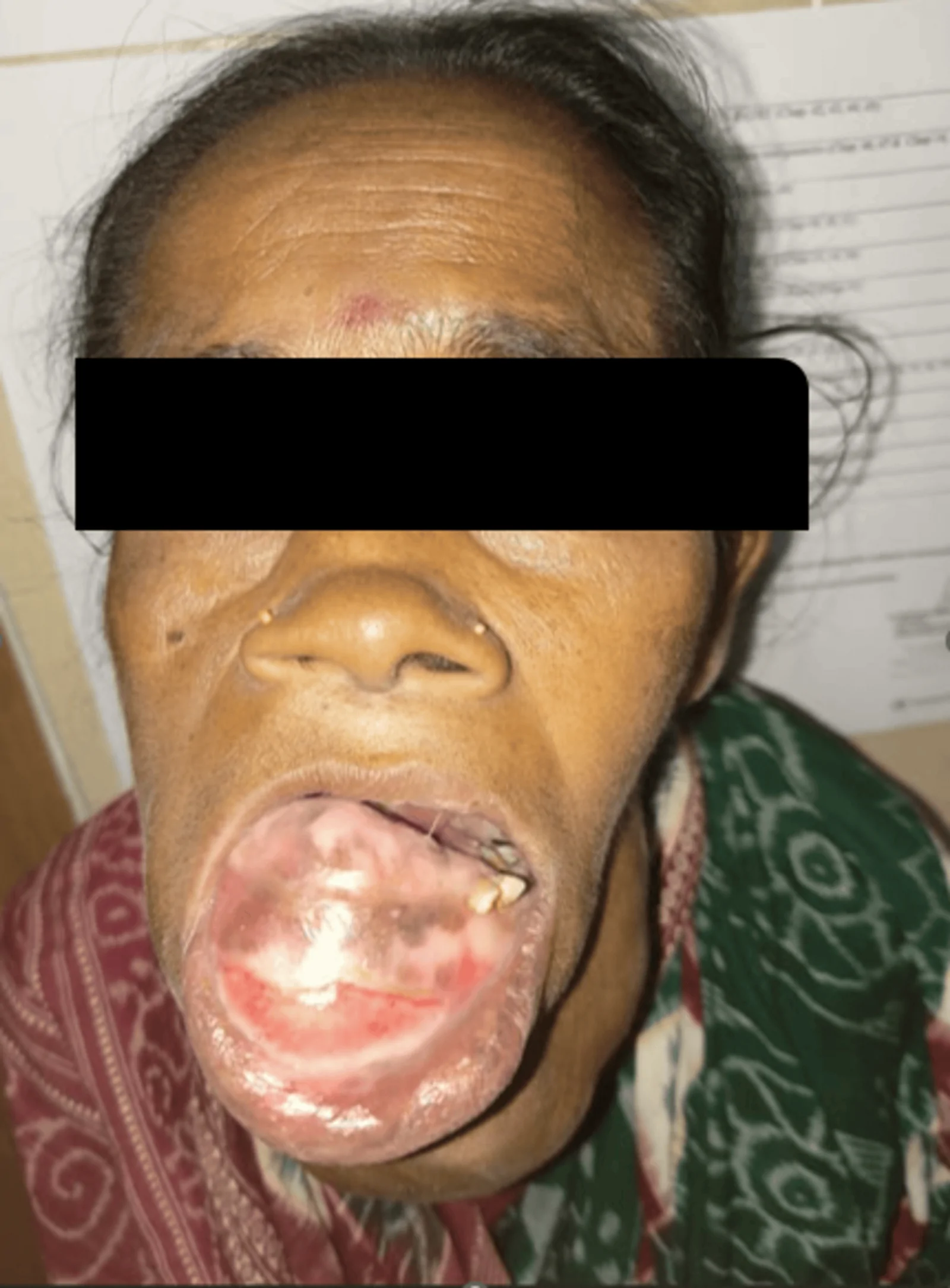
Preoperative image of the patient showing the large mandibular growth.

Enhanced computed tomography revealed a massive 13 × 11 × 15 cm expansile lytic lesion involving the mandible with extensive intralesional calcifications, bicortical expansion, cortical thinning, and cortical breaches. The lesion extended from the right ascending ramus to the left mandibular angle, as shown in Figure 2 (coronal cut) and Figure 3 (sagittal cut). Radiological findings strongly suggested calcifying epithelial odontogenic tumor. Three-dimensional reconstruction, as shown in Figure 4, shows both anterior and lateral views for better understanding of the extent of the tumor. 

**Figure 2:**
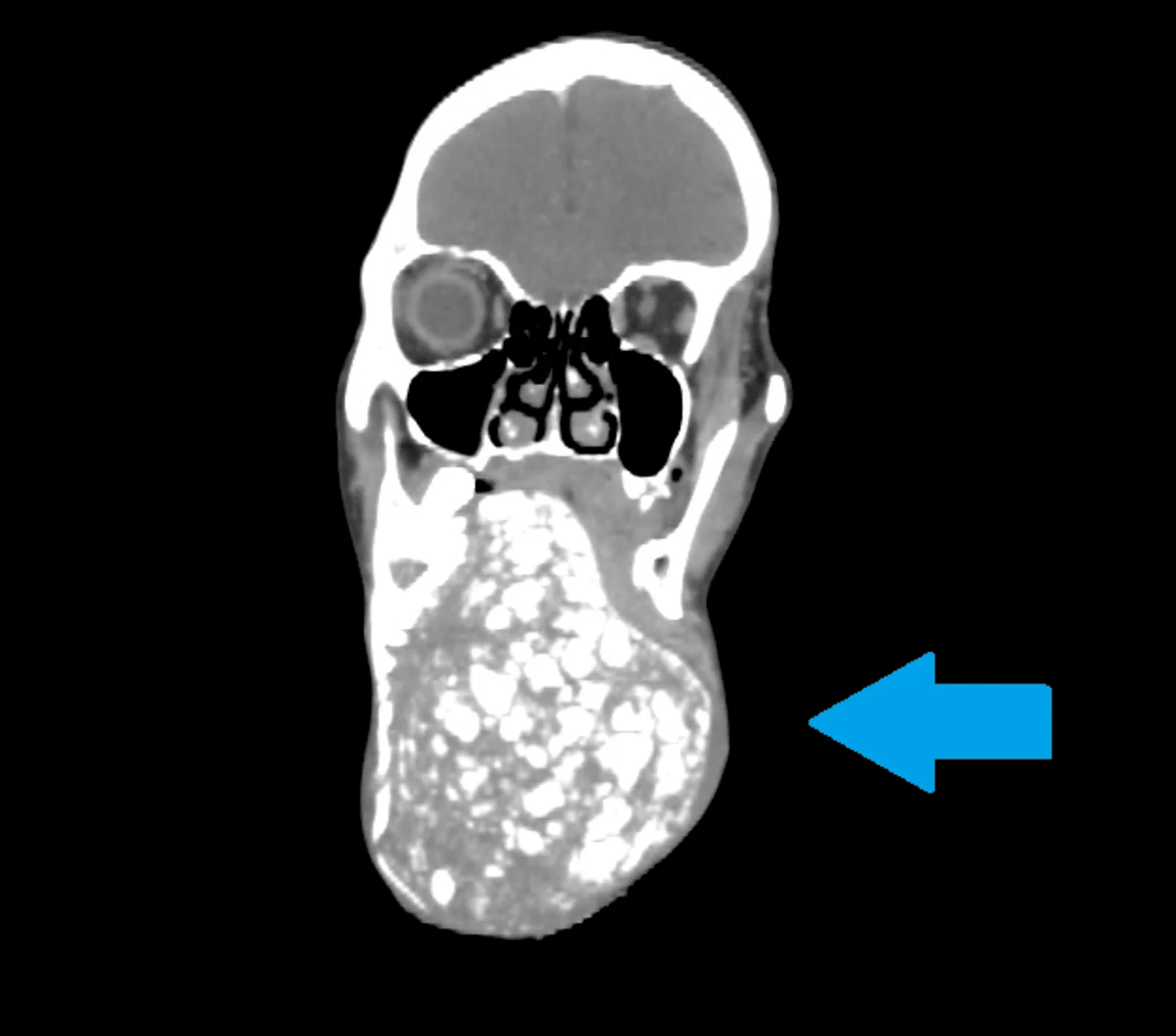
Contrast-enhanced computed tomography (coronal cut) showing an expansile mandibular mass with cortical thinning (blue arrow). The large expansile lesion 13 x 11 x 15 cm (AP x TR x CC) centered in the central mandible extends up to the ramus on the right side and the posterior mandibular body on the left, causing extensive mandibular buccolingual and anterioposterior expansion, with large floor of mouth involvement.

**Figure 3:**
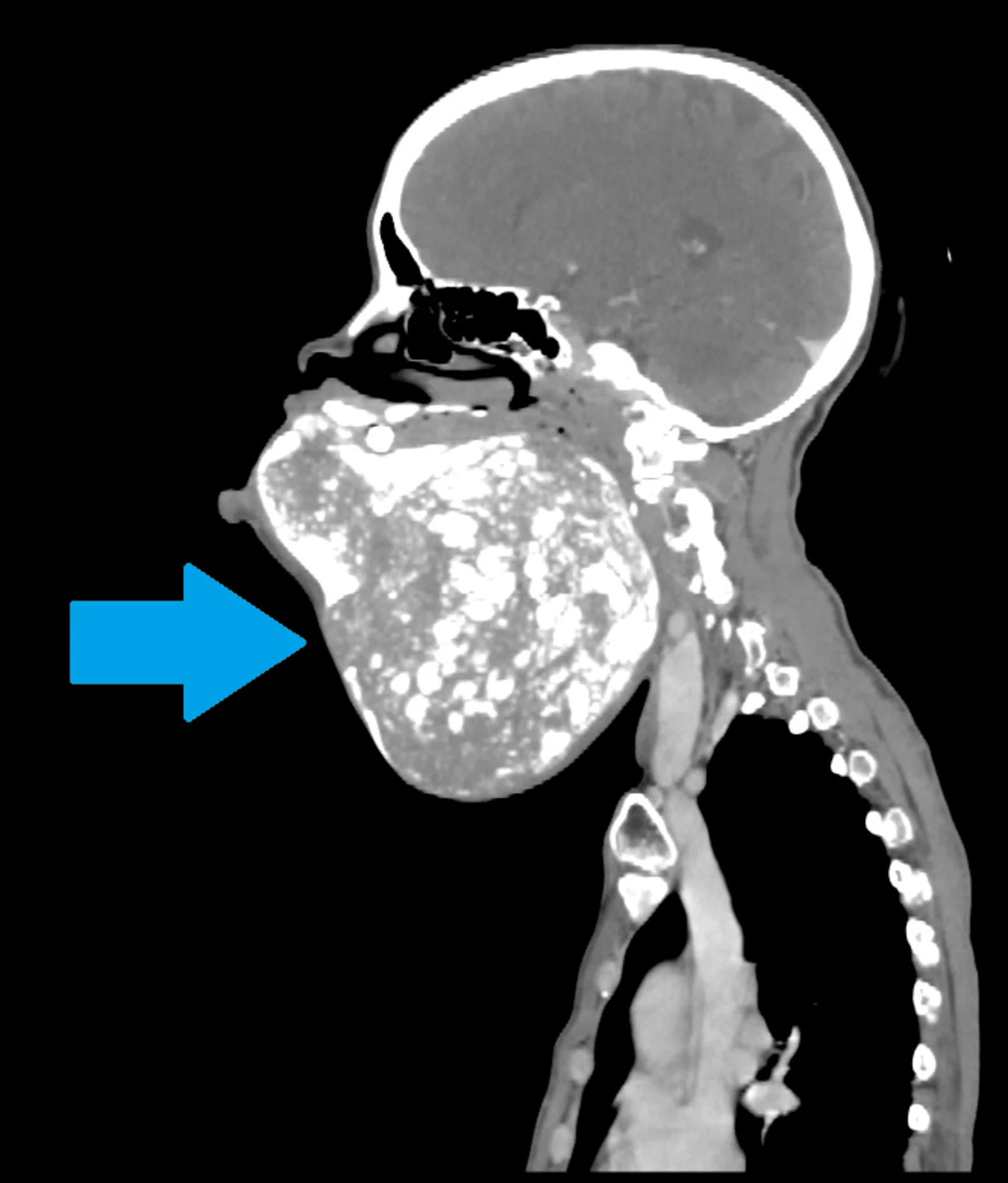
Contrast-enhanced computed tomography of the face and neck saggital cut. The site is shown by the blue arrow. Saggital cuts showing numerous intralesional heavily calcifiednodules are noted studded within the mandible. Multifocal areas of cortical thinning and focal cortical breaches seen.

**Figure 4:**
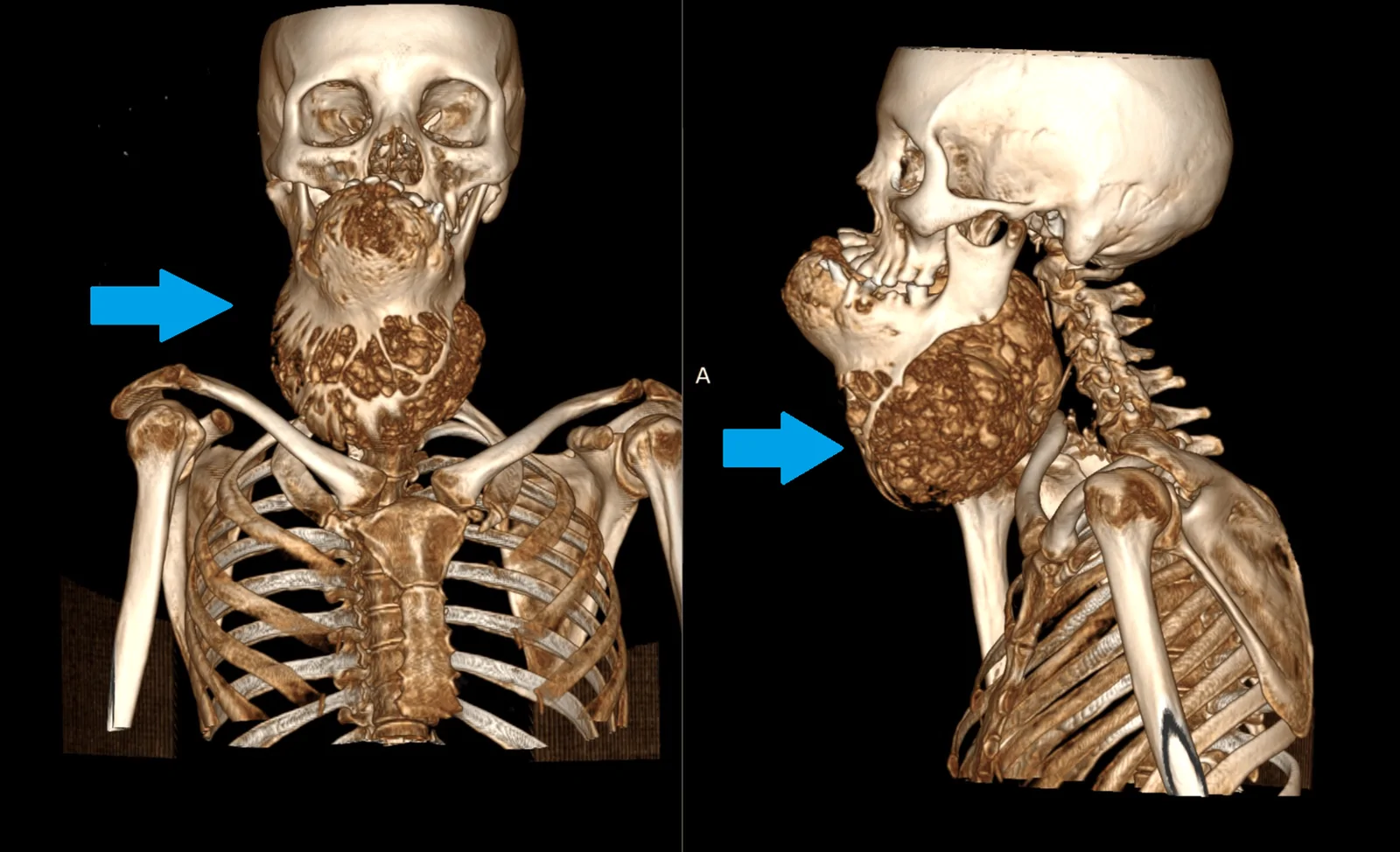
Three-dimensional reconstruction of the tumor for better understanding of the extent of the tumor pointed by blue arrows.

Incisional biopsy demonstrated fibrocollagenous tissue with areas of dead bone and was inconclusive for definitive diagnosis. However, due to the extensive mandibular involvement and aggressive radiological appearance, provisional diagnosis favored calcifying epithelial odontogenic tumor. The patient was planned for a subtotal mandibulectomy in view of difficulty in feeding and facial deformity.

After optimization and explaining the risks of the surgery, the patient was taken up for subtotal mandibulectomy with preservation of bilateral condyles and upper rami. The patient was placed in supine position with adequate neck extension. A vertical incision was marked (Figure 5). 

**Figure 5:**
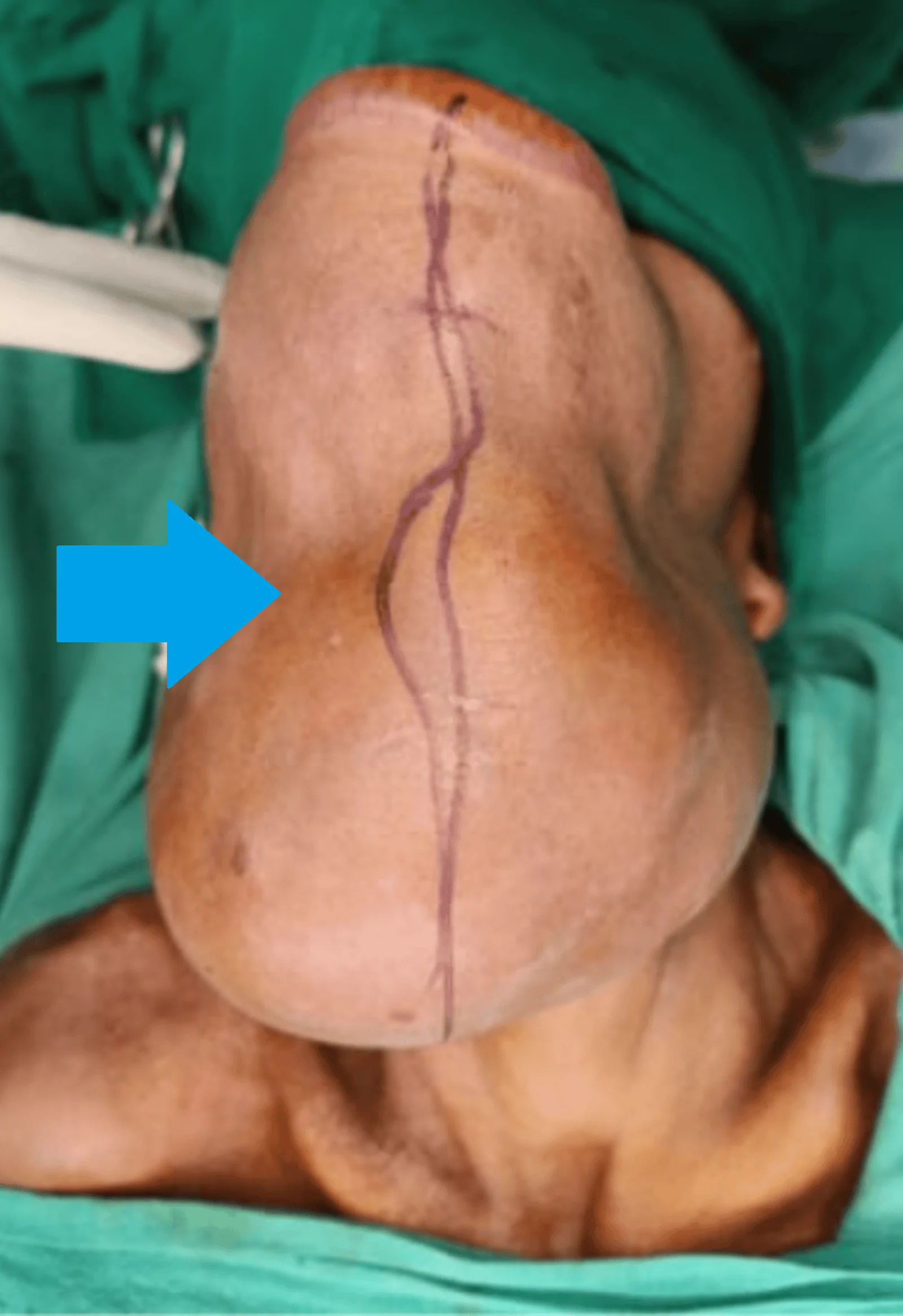
Incision line marked (blue arrow) over the mandibular growth after intubating the patient by nasal intubation. Incision line extending proximally from the lip to almost a point below the growth distally.

The incision was then deepened in layers, and on either side, flaps were raised adequately in order to completely expose the growth and the mandible (Figure 6). After carefully dissecting and raising the flaps on either side, both the rami of the mandible were exposed. Left-side mandibulotomy was done, preserving the upper ramus and condyle. The right-side ramus was found to be involved up to the mid ascending ramus. Right-side mandibulotomy preserving part of the ramus and condyle. 

**Figure 6:**
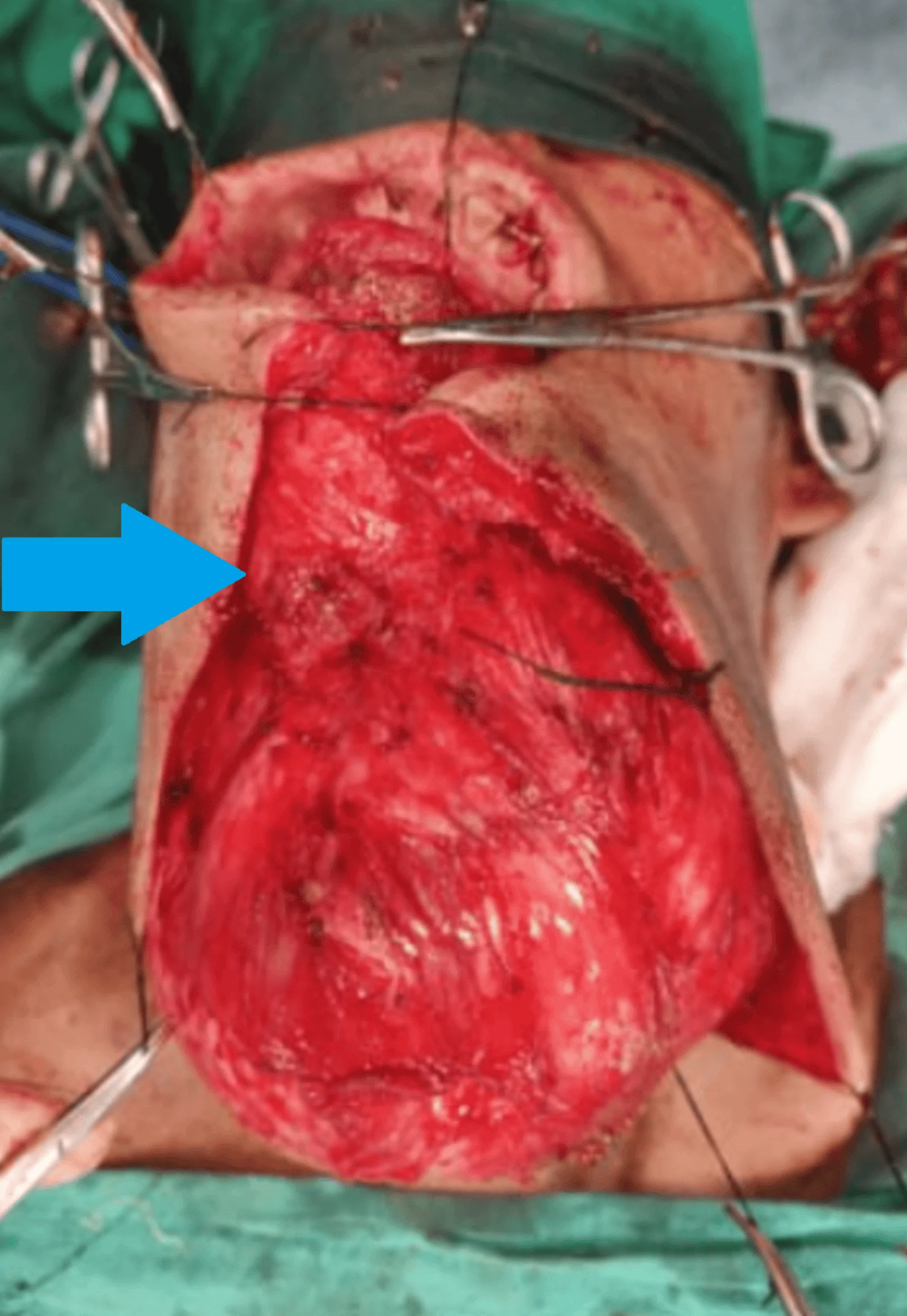
Incision was deepend and falps were raised on either side exposing the mandibular mass shown by the blue arrow.

The tumor was completely dissected from the floor of the mouth, the gingivolingual sulcus, and muscles of the upper neck. The specimen was removed (Figure 7). Reconstruction was performed using a titanium reconstruction plate with local flap coverage (Figure 8). The buccal mucosa was sutured to the inferior aspect of the tongue. Floor of mouth reconstruction was done using a local flap. Elective tracheostomy was performed to secure a stable airway during the postoperative period.

**Figure 7:**
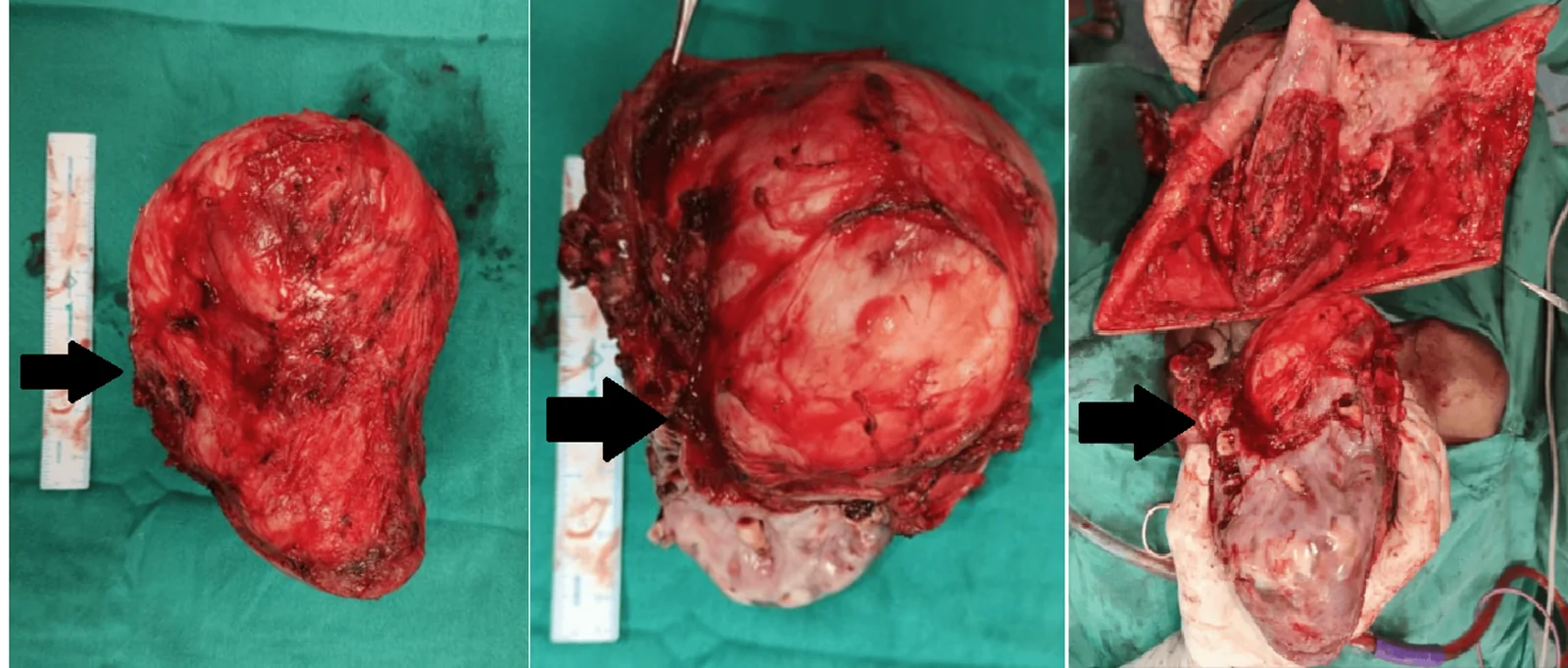
Resected specimen showing the mandibular growth after subtotal mandibulectomy. The black arrow showing the specimen (mandibular mass). The first image shows the posterior aspect of the mandibular mass. The second image shows the anterior aspect of the mandibular mass. In the third image, the specimen is placed close to the resected ends for better understanding of the orientation of the specimen.

**Figure 8:**
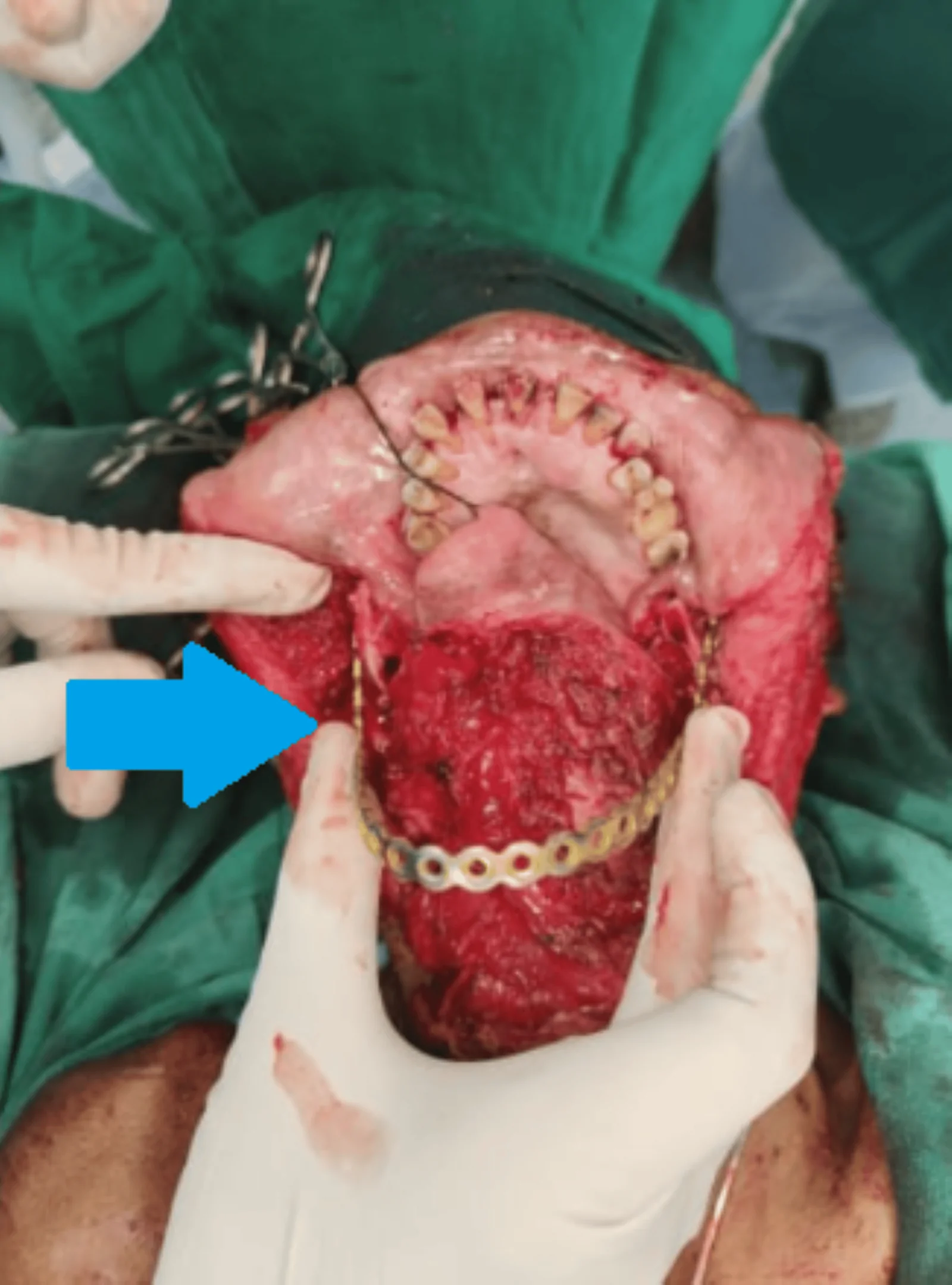
Reconstruction with a titanium plate (blue arrow) after subtotal mandibulectomy.

The final histopathological examination revealed fibro-osseous proliferation composed of irregular immature woven bony trabeculae within fibrous stroma without evidence of odontogenic epithelial islands, amyloid deposition, or Liesegang ring calcifications, confirming fibrous dysplasia (Figure 9).

**Figure 9:**
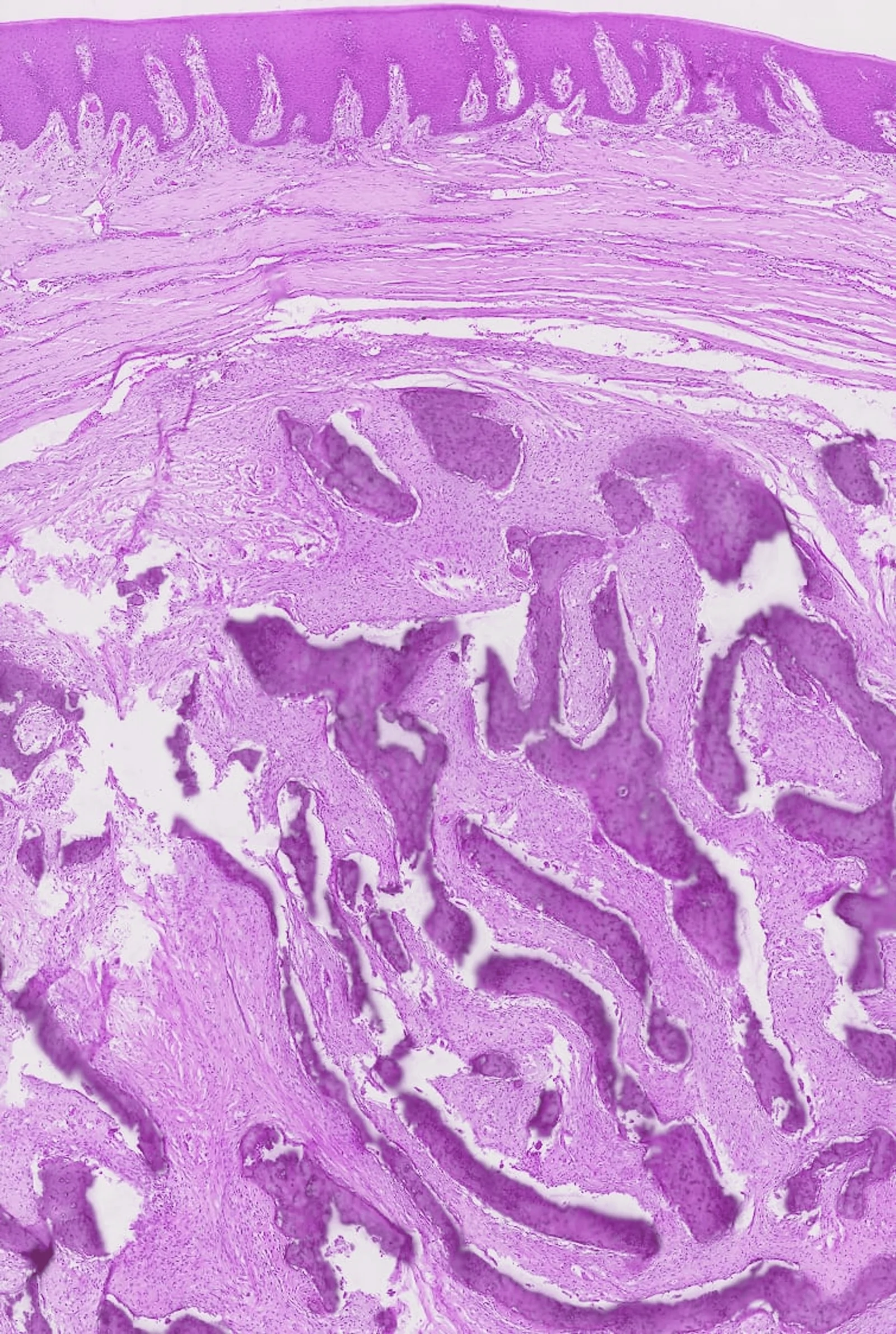
Histopathological image of the resected mandibular mass. 10x hematoxylin and eosin (H&E) stain: Oral mucosa lined by straitified squamous epithelium with underlying fibrosseous lesion showing irregular bony trabeculae surrounded by fibrocellular stroma more suggestive of a fibrous dysplasia.

Table 1 shows the patient's entire timeline. 

**Table 1:** Patient's timeline.

Chief complaints	Progressive jaw swelling for four years, neck pain and swelling, progressive difficulty in mouth opening for three months, and inability to eat due to severe trismus.
Clinical findings	Examination revealed a giant mandibular swelling with marked facial deformity and severe restriction of mouth opening, resulting in poor oral intake. No clinically significant cervical lymphadenopathy was identified.
Operative procedure	Subtotal mandibulectomy with titanium plate reconstruction with tracheostomy.
POD 1	Shifted to the surgical ICU in view of post-operative care.
POD 2	Patient was extubated and was shifted to ward in view of post-operative care.
POD 5	Removed closed wound suction device.
POD 9	Tracheostomy was decannulated and removed. Speech and swallow exercised along with stoma care was given.
POD 12	Complete suture removal was done and patient was discharged.

## Discussion

CEOT is a rare odontogenic neoplasm commonly reported in adults between 40 and 60 years of age [4,5]. The lesion frequently involves the posterior mandible and may demonstrate a mixed radiolucent-radiopaque appearance with intralesional calcification. Histopathologically, CEOT is characterized by sheets of polyhedral epithelial cells, amyloid-like material, and Liesegang ring calcifications. On the other hand, fibrous dysplasia is a benign fibro-osseous lesion resulting from the replacement of normal bone with immature woven bone within fibrous stroma, as seen on the slide picture (Figure 9).

In general, fibro-osseous lesions of the jaw are a group of benign disorders that can affect the bone. Fibrous dysplasia was coined for the first time by Lichtenstein and Jaffe in 1942 [6]. In this condition, bone tissue is replaced by irregular fibrous tissue between normal and immature bone. Its etiology remains unknown, with an incidence of 3% of all bone tumors. Coming to benign bone tumors, they constitute roughly 5-7% [6]. In the head and neck region, fibrous dysplasia is seen involving the maxilla, mandible, and frontal bone in decreasing order. Common symptoms of fibrous dysplasia mainly include pain, facial deformity, fractures, and dental and neurological impairments [6]. The diagnosis of fibrous dysplasia poses a challenge. It requires a combined effort of radiological, clinical, and histopathological characteristics. The gold standard is always histopathological [6]. 

Radiologically, CT imaging is required to determine the extent of disease. One of the major findings is a thin cortex without distinct borders. It may present in three ways radiologically: one being a ground-glass pattern (56%), second being a dense pattern (23%), and lastly the cystic pattern (21%) [7]. However, in progressive disease or a lesion slowly growing, it may have both radiopaque and radiolucent mixed lesions, which may not be very characteristic, as in our case.

As seen in the present case, there were features of cortical thinning with scattered calcified features similar to that of CEOT. However, as it was a slow-growing fibrous dysplasia, it had both mixed radio-opaque and radiolucent features, as mentioned above. 

In the present case, the lesion demonstrated extensive calcification, cortical thinning, cortical breaches, and massive mandibular expansion, all of which strongly mimicked advanced CEOT radiologically. Initial biopsy was inconclusive and failed to establish a definitive diagnosis. The final histopathological examination of the resected specimen confirmed fibrous dysplasia by demonstrating fibro-osseous proliferation with irregular woven bony trabeculae within fibrous stroma without odontogenic epithelial components. Despite the final benign histopathological diagnosis, aggressive surgical management was considered necessary because of the lesion’s massive size, severe facial deformity, nutritional compromise, trismus, cortical destruction, and inability to confidently exclude aggressive odontogenic neoplasm preoperatively. Subtotal mandibulectomy with reconstruction plate fixation and local flap reconstruction provided satisfactory structural rehabilitation.

In our case, we used a titanium plate, but other reliable options are stainless steel and Vitallium. Initially, Vitallium was being used, but due to its low malleability, it can make application very difficult. AO stainless steel-plated and AO titanium reconstruction plates were developed as a fast, single-staged, and reliable alternative [8]. THORP (titanium hollow osseointegrated reconstruction plate) is a reconstruction plate system that uses perforated hollow titanium screws that allow more bone in-growth, thereby increasing bone stability [8].

This case highlights the importance of correlating clinical, radiological, and definitive histopathological findings in giant mandibular lesions with overlapping imaging features.

Fibrous dysplasia should be differentiated from other fibro-osseous and sclerotic lesions of the jaws based on clinical presentation, imaging characteristics, and histopathological findings. The principal differential diagnoses include ossifying fibroma, cemento-osseous dysplasia, osteoma, chronic sclerosing osteomyelitis, Paget disease of bone, osteosarcoma, and, less commonly, osteoblastic metastases [9].

## Conclusions

Giant fibro-osseous lesions of the mandible may closely mimic calcifying epithelial odontogenic tumors radiologically, particularly when associated with extensive calcification and cortical destruction. Accurate diagnosis requires careful clinicoradiological correlation and definitive histopathological evaluation. Surgical resection with appropriate reconstruction remains an effective treatment strategy for extensive mandibular lesions causing significant functional and cosmetic compromise.

## References

[1] Philipsen HP, Reichart PA (2000). Calcifying epithelial odontogenic tumour: biological profile based on 181 cases from the literature. Oral Oncol.

[2] PI JJ (1958). A calcifying epithelial odontogenic tumor. Cancer.

[3] Franklin CD, Pindborg JJ (1976). The calcifying epithelial odontogenic tumor. A review and analysis of 113 cases. Oral Surg Oral Med Oral Pathol.

[4] Neville BW, Damm DD, Allen CM, Chi AC (2016). Oral and Maxillofacial Pathology. 4th ed.. Oral and Maxillofacial Pathology. 4th ed. St Louis: Elsevier.

[5] (2005). World Health Organization Classification of Tumours: Pathology and Genetics of Head and Neck Tumours. https://scholar.google.com/scholar?q=intitle:World%20Health%20Organization%20Classification%20of%20Tumours%3A%20Pathology%20and%20Genetics%20of%20Head%20and%20Neck%20Tumours.

[6] Yáñez F, Morales D, Arriagada A, Pinedo FJ (2021). Craniofacial fibrous dysplasia of the mandible: a case report and review of current literature. Res Rep Oral Maxillofac Surg.

[7] Obermeier KT, Hartung JT, Hildebrandt T (2023). Fibrous dysplasia of the jaw: advances in imaging and treatment. J Clin Med.

[8] Kumar BP, Venkatesh V, Kumar KA, Yadav BY, Mohan SR (2016). Mandibular reconstruction: overview. J Maxillofac Oral Surg.

[9] Van Hoe S, Bladt O, Van Der Steen K, Van den Eynde H (2021). Sclerotic lesions of the jaw: a pictorial review. J Belg Soc Radiol.

